# Improvement of Nutritional Status among Tuberculosis Patients by *Channa striata* Supplementation: A True Experimental Study in Indonesia

**DOI:** 10.1155/2020/7491702

**Published:** 2020-01-11

**Authors:** Isa Ma'rufi, Khaidar Ali, Sigit Kusuma Jati, Anik Sukmawati, Kurnia Ardiansyah, Farida Wahyu Ningtyias

**Affiliations:** ^1^Department of Environmental Health and Occupational Safety, School of Public Health, University of Jember, Jember Regency 68121, Indonesia; ^2^Department of Health Behavior, Environment and Social Medicine, Faculty of Medicine, Universitas Gadjah Mada, Yogyakarta 55281, Indonesia; ^3^Jember Chest Hospital, 68118 East Java, Indonesia; ^4^Department of Nutritional Health, School of Public Health, University of Jember, Jember Regency 68121, Indonesia

## Abstract

**Objective:**

To analyze the effect of *Channa striata* supplementation on body mass index among tuberculosis patients, in which their health status is also investigated.

**Methods:**

This study employed a true experiment. The study was designed randomized pretest-posttest with a control group, in which 200 respondents were enrolled. Body mass index (BMI), used as a nutritional status indicator, was measured every week for a month. Chi-square test was used to analyze the data with a significance level of 5% by STATA 13.

**Results:**

The mean BMI of all groups increases during the month, in which rapid alteration occurs in the treatment group. The mean BMI (kg/m^2^) in the treatment group at weeks 0–4 was reported to be 17.43, 17.65, 17.90, 18.04, and 18.22, respectively. Meanwhile, the mean BMI (kg/m^2^) at weeks 0–4 in the control group was reported to be 17.20, 17.36, 17.57, 17.71, and 17.96, respectively. Furthermore, the alteration from severe thinness to higher BMI level in the treatment group is the highest. Based on the statistical test, there were no differences in BMI between the treatment and control groups (*p* > 0.05). However, the alteration of nutritional status in the treatment group is faster than that in the control group. In addition, there is no difference in their health status between the treatment and control groups (*p* > 0.05), except vomiting (*p* < 0.05).

**Conclusion:**

The BMI among tuberculosis patients with *Channa striata* supplementation is increasing faster than that in the control group within a month with a minimum potential negative effect.

## 1. Introduction

Tuberculosis (TB), caused by *Mycobacterium tuberculosis*, has become an emerging disease since 1992, where the morbidity and mortality rate is high. TB has infected about one-fourth of the world's population [1]. The World Health Organization (WHO) [2] also reported that 10 million people are suffering from TB in 2017, which causes 1.6 million deaths among the population and 230,000 deaths among children [2]. The incidence of TB is high in developing countries such as Africa and Asia [3] where two-thirds of cases in 2017 were contributed by India, China, Indonesia, the Philippines, Pakistan, Nigeria, Bangladesh, and South Africa [4]. Therefore, most developing countries suffer from double burden disease.

The existence of multidrug-resistant tuberculosis (MDR-TB) is also threatening for the population where the survival rate is 9 years with poor treatment and the resistant bacteria spread along that time [5]. The study of MDR-TB is well documented. Gaborit et al. found that the incidence of MDR-TB in a low-incidence area is correlated with migration from high-risk countries [6], where previously used second-line drug is a risk factor, involving extensive drug-resistant (XDR) tuberculosis [7]. MDR-TB is caused by several risk factors, namely, self-motivation, awarness, counselling‐ , family‐ , social‐ , and nutritional‐ support [8]. Moreover, the incidence of MDR-TB is high worldwide, which is correlated with the high prevalence of HIV, global poverty, and emergence of immense drug-resistant tuberculosis [9].

*Mycobacterium tuberculosis* spreads to a person through air [1, 10]. Besides the lung, the bacteria also attack skin, skull, brain, gut, and kidney [11], which leads to disability [12]. TB is commonly found in tropical and subtropical countries. Socioeconomic aspects such as urbanization, poverty, crowd, and low sanitation are the risk factors for TB among the population [13, 14]; malnutrition is also a risk factor for TB that increases morbidity and mortality rate in acute and chronic disease, particularly among children [15–18]. Severe malnutrition leads to a higher mortality rate [19], in which malnutrition causes secondary immunodeficiency among TB patients that elevates host vulnerability toward infection, particularly tuberculosis [20, 21]. Moreover, poor nutritional status among TB patients may suppress cell‐mediated immunity, that is, the principle host defense against TB [22, 23].

Both *M. tuberculosis* infection and progressivity of *M. tuberculosis* are tuberculosis risk factors [23–25]. TB leads to reduction in appetite, nutrient malabsorption, macro- and micronutrient malabsorption, and also wasting metabolism [21], which causes micro- and macro-nutrient deficiency among patients [23, 25, 26]. TB patients are reported to have low level of hemoglobin, retinol, and zinc [27]. Hence, TB coinfection with HIV worsened through severe micronutrient malnutrition and wasting [28, 29]. Lönnroth et al. reported that BMI is associated with tuberculosis [26], where early death among TB patients with BMI <17 kg/m^2^ is high [30]. On the other hand, higher BMI level reduces mortality among TB patients [31], and people with obesity have a lower risk of active pulmonary tuberculosis [32]. Therefore, improving nutritional status is important to manage tuberculosis.

Indonesia is a tuberculosis-endemic area, where tuberculosis patients suffered from malnutrition (BMI < 18.5 kg/m^2^), lived in a crowded area, and were unemployed [13]. In 2017, the WHO estimated that TB incidence in Indonesia is 446,732 cases [33]. However, 425,089 cases were reported by the Health Ministry of Indonesia [34], which is higher than that reported in 2016 with 351,893 cases [35]. In 2018, the TB incidence in Indonesia is reported to be 321 per 100,000 populations [36]. East Java, one of the biggest provinces in Indonesia, also has high tuberculosis cases. In 2017, the tuberculosis case in East Java is the highest after West Java with 48,323 cases. The highest tuberculosis case in East Java occurs in Surabaya Municipally followed by Jember District with 2,153 cases in 2016 [37]. In addition, tuberculosis case in Situbondo is reported to be 595 cases [37].

The objective of the study is to analyze the effect of *Channa striata* supplementation on body mass index among tuberculosis patients. The authors also investigate their health status after *Channa striata* extract administration.

## 2. Materials and Methods

### 2.1. Study Area and Time

This study was conducted in Jember District and Situbondo District, East Java, Indonesia, which is approximately 150–199 km from the capital city of East Java province. Based on Statistics Office, Jember and Situbondo had 31 subdistricts and 13 subdistricts, respectively [38, 39]. In addition, this study was held on June–December 2017.

### 2.2. Method and Study Design

**Figure 1:**
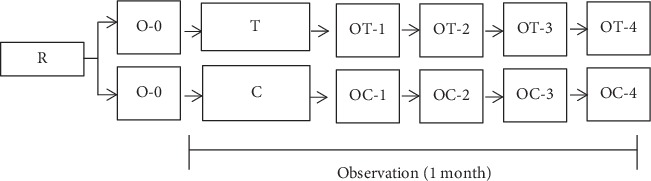
The study design. Description: R: randomization; O-0: observation at week 0; T: treatment group; C: control group; OT-1–4: observational treatment group at weeks 1–4; OC-1–4: observational control group at weeks 1–4.

This study employed a true experiment, in which the study design was a randomized pretest-posttest control group design (Figure 1). Randomization was used to divide the samples into two groups, namely, treatment and control groups, where the intervention is *Channa striata* supplementation. Moreover, the control group that was given placebo was used for comparison. The extract of *Channa striata* and placebo were administered 3 times a day for 1 month. The authors then evaluated the effect of *Channa striata* supplementation among respondents.

*Channa striata* extract or supplement was permitted for consumption by the Health Ministry of Indonesia (Registered number: P-IRT: 202350901620); 500 mg of *Channa striata* supplement contains 90% extract of *Channa striata* and 10% others (protein (80.9%), albumin (12.5%), and polyphenol bioflavonoid (6.6%)) [25]. In this study, the treatment was performed on an ambulatory basis, and field research assistant was used to monitor and to supervise respondents to take *Channa striata* extract and placebo regularly during the study. Respondents were also asked about their health status after performing treatment.

Body mass index (BMI) was used to evaluate the effect of *Channa striata* extract among respondents. The systematic review from Lönnroth et al. was used as a reference in this study [26], where BMI is appropriate to measure the nutritional status [26]. The calculation and classification about BMI are referred to as the WHO. The BMI calculation is as follows [40]:

(1) $$\begin{array}{c} \text{BMI}=\frac{\text{weight }\left(\text{kg}\right)}{{\text{height}}^{2}\;{\left(\text{m}\right)}^{2}}. \end{array}$$

Based on the WHO, BMI was classified into 4 categories [40]: (1) underweight, (2) normal, (3) overweight, and (4) obese. However, there were 3 categories used in this study, namely [40], (1) underweight (severe thinness: <16 kg/m^2^, moderate thinness: 16–16.99 kg/m^2^, and mild thinness: 17–18.49 kg/m^2^); (2) normal: 18.5–24.99 kg/m^2^; and (3) overweight: ≥25 kg/m^2^. Hence, the criteria were comprehensive and specific among tuberculosis respondents.

The BMI data (weight and height; kg/m^2^) were collected weekly for a month (weeks 0, 1, 2, 3, and 4) by nurse in primary healthcare center at Jember and Situbondo. In addition, health status among respondents after *Channa striata* extract administration was asked by field research assistant at week 2. The questions asked were about allergy, rash, inhale nuisance, nausea, vomiting, diarrhea, smooth inhale, and good appetite, and the responses were none, mild, moderate, and severe or high.

### 2.3. Population and Sampling

The population was new pulmonary TB patients (sputum-smear positive TB), and the study was conducted with TB standard treatment with antibiotics where the prescription referred was first-line TB drugs (isoniazid, rifampicin, ethambutol, streptomycin, and pyrazinamide). The total population was reported to be 2,733 (Jember: 2,176 patients and Situbondo: 557 patients). The total number of samples in this study was 200 respondents, where the distribution in the treatment and control groups was 103 respondents and 97 respondents, respectively. The exclusion criteria were as follows: (1) MDR-TB, (2) HIV/AIDS, (3) diabetes mellitus, and (4) failure tuberculosis treatment. Hence, respondents who did not take tuberculosis medicine and/or *Channa striata* extract regularly will be dropped out.

Participants voluntarily joined this study. After the respondents were informed about the study, they had signed informed consent, and respondents can withdraw from participation. Moreover, a pulmonary specialist doctor also monitored respondents after *Channa striata* extract administration.

### 2.4. Data Analysis

The authors employed STATA 13 (College Station, Texas) to analyze the data. After data checking and cleaning, descriptive analysis was performed where findings were presented with tabulation that contains frequency, percentage, mean, standard deviation, and confidence interval. Furthermore, chi-square test was performed to examine the effect of *Channa striata* supplementation on BMI among respondents (treatment and control groups). There were missing data on health status among respondents. The authors then exclude these missing data from the analysis. Meanwhile, Wilcoxon signed-rank test was also conducted to analyze the effect before and after treatment. The significant level was 5% (*α* = 0.05) with 95% confidence interval.

## 3. Results and Discussion

### 3.1. Respondent Characteristics

Table 1 presents the characteristics of respondents. Based on age, the number of respondents >50 years of age is the highest with 37.5%, where it is similar to the distribution by group. The total number of male respondents in all groups is higher than that of female respondents, whereas the number of female respondents is high in the treatment group with 52.43%. In addition, most of the respondents are not well educated (none and elementary) with a proportion of 56.50%. Based on the statistical test, the *p* value of all variables is reported to be >0.05. This indicates that the distribution of respondents is similar in the treatment and control groups.

### 3.2. Health Status

Table 2 presents health status among respondents at week 2. In the treatment group, more than 75% of respondents did not report rash, inhale nuisance, vomiting, and diarrhea, and 61.70% and 58.51% of respondents also did not report allergy and nausea, respectively. In addition, 42.55% and 39.36% of respondents got mild smoothing inhale and good appetite, respectively. Meanwhile, more than 75% of respondents not reported rash, vomiting, and diarrhea in the control group. Moreover, 43.96% and 34.07% of respondents got mild level of smoothing inhale and good appetite.

Based on the chi-square test, the health status between the treatment and control groups is similar (*p* > 0.05), except vomiting (*p* < 0.05).

### 3.3. *Channa striata* Supplementation and Body Mass Index

The effect of *Channa striata* supplementation on body mass index (BMI) at weeks 0–4 is shown in Table 3. The distribution of BMI among respondents at week 0 is similar in the treatment and control groups (*p* > 0.05), whereas 38.83% and 30.93% of respondents are classified into severe thinness among the treatment and control groups, respectively. In addition, the distribution of normal BMI in the control group at week 0 is higher than that in the treatment group in this study.

In the treatment group, the distribution of respondents classified as normal at weeks 1–4 is reported to be 29.13%, 30.10%, 33.98%, and 37.86%, respectively. On the other hand, the distribution of respondents classified as normal at weeks 1–4 among the control group is 35.05%, 39.18%, 41.24%, and 41.24%, respectively. Furthermore, based on the statistical test, there is no difference in BMI between the treatment and control groups at weeks 1–4 (*p* > 0.05).

Table 4 presents the mean BMI in the treatment and control groups at weeks 0–4. The mean BMI in the treatment group at weeks 0–4 is reported to be 17.43 kg/m^2^, 17.65 kg/m^2^, 17.90 kg/m^2^, 18.04 kg/m^2^, and 18.22 kg/m^2^, respectively. These data showed that the mean BMI among respondents in the treatment group increased by 0.79 kg/m^2^ from weeks 0 to 4. Meanwhile, the mean BMI in the control group at weeks 0–4 is reported to be 17.20 kg/m^2^, 17.36 kg/m^2^, 17.57 kg/m^2^, 17.71 kg/m^2^, and 17.96 kg/m^2^, respectively. Based on the statistical test, *p* value in both the treatment and control groups is <0.05. It showed that there is difference in BMI between before and after treatment.

Tuberculosis becomes a great challenge worldwide, particularly in developing countries. Not only the high incidence of TB, but also the existence of MDR-TB and XDR-TB is threatening population, in which MDR-TB patients are affected by improper standard tuberculosis treatment or reinfection [41]. However, MDR-TB patients are also newly infected by first-line drug-resistant bacteria. Socioeconomic aspects, lack of transportation cost and of social support, and also poor communication of health worker lead to nonadherence of TB treatment [42–44]. In addition, 6-month duration of standard TB treatment also leads to the adherence of TB recovery among patients. Therefore, nutritional approach is needed to accelerate the duration of treatment without reducing the quality to overcome tuberculosis cases worldwide, in which case this study used *Channa striata* extract as additional nutritional supplement.

Based on Table 1, the total number of respondents aged >50 years in all groups is the highest with 37.50% followed by respondents aged 40–49 years (21.50%). Furthermore, tuberculosis case is also high among reproductive age group (20–29 years) with 18.5%. The total male patients are documented higher than the female patients in this study with 52.0%. Based on education level, tuberculosis patients have lower education level with 56.6% (none and elementary). On the other hand, only 1.50% of tuberculosis cases have high education level. Moreover, the distribution of respondents according to age, sex, and educational level is similar in the treatment and control groups (*p* > 0.05). The similar distribution of respondent characteristic between the treatment and control groups prevents bias.

All populations are at risk of tuberculosis infection where TB infects most vulnerable population such as old person and cancer [45], HIV, and diabetes patients and close contact, family history, poverty, overcrowding, and alcohol use are also factors for TB infection [46, 47]. Moreover, Popovic et al. in their review also found that PM10, nitrogen dioxide, and sulfur dioxide were correlated to TB although it is inconsistent [48]. TB cases among older respondents in this study are high, where aging causes dysregulation in immune function that may cause vulnerability for TB infection [49]. The education level among TB patients in this study is similar to that reported in the study of Kirenga et al. where major TB cases occur in people with low education level (none and primary education) with 35.3% [46].

The health status among TB patients is shown in Table 2. Based on Table 2, up to 79% of respondents not reported rash, inhale nuisance, vomiting, and diarrhea after *Channa striata* extract administration. Furthermore, up to 39% of respondents got mild smoothing inhale and good appetite feeling. In the control group, up to 79% of respondents not reported rash, vomiting, and diarrhea. In addition, up to 34% of respondents got mild smoothing inhale and good appetite feeling. Therefore, the health status among the treatment group is high, although the health status among the treatment and control groups is similar or not significant (*p* > 0.05), except vomiting (*p* < 0.05).

Body mass index (BMI) is commonly used to measure the nutritional status among the population. BMI is also widely used as a risk factor for several health issues worldwide [50], and higher BMI is a protective factor against tuberculosis [51]. The effect of *Channa striata* supplementation on body mass index is shown in Table 3. The distribution of BMI among respondents is high in severe thinness with 35% at week 0, whereas the total number of respondents with severe thinness in the treatment group is higher than that in the control group with 38.83%. However, statistical test at week 0 showed nonsignificant result (*p* > 0.05).

There is an alteration in BMI in both groups between weeks 0–4, in which the distribution of total respondents with severe thinness is descending at weeks 1–4 with 31%, 28%, 27%, and 23.5%, respectively. However, the alteration (BMI at week 4–BMI at week 0) of severe thinness in the treatment group is higher than that in the control group, where 13.59% of respondents with severe thinness among the treatment group at weeks 0–4 moved to higher BMI criteria. Meanwhile, the alteration of respondents with severe thinness in the control group at weeks 0–4 is reported to be 9.28%. Based on Table 4, the alteration mean BMI in the treatment group at weeks 0–4 is higher than that in the control group, in which the alteration mean BMI in the treatment group during the study is 0.79 kg/m^2^ compared to the control group with 0.76 kg/m^2^. Therefore, the alteration rate in the treatment group is faster than that in the control group within a month, where statistical test showed significant (*p* < 0.05).

The WHO issued END TB strategy to manage tuberculosis incidence worldwide [52], in which the trends of TB prevalence are relatively steady even elevate in some regions. TB patients suffer from malabsorption, which causes not only micro- and macro-nutrient deficiency, but also vitamin deficiencies [53]. Vitamin D deficiency is a risk factor for the development of active TB [54], where tuberculosis can be controlled by vitamin D supplementation [54]. Therefore, management of nutritional status such as routinely providing either free food or energy supplements may improve antituberculosis treatment response [55, 56], particularly in underdeveloped area [21, 22].

The study from Abba et al. [18] and Gupta et al. [21] showed the effect of supplementation of zinc, arginine, copper, iron, vitamins A, C, D, and E, and their combination on tuberculosis patients. In addition, there is a significant alteration of BMI after tuberculosis patients are administered macronutrients, where the BMI of the treatment group is higher than that of the control group [57].

*Channa striata* extract is a potential supplement that elevates the level of total amino acid, arginine, and leucine serum [58]; *Channa striata* extract contains 17 amino acids, and the major amino acids are glutamate, lysine, leucine, asparagine, Alani, arginine, and valine [59]. The largest fraction of *Channa striata* extract is albumin (64.61%), and glucose, zinc, Cu, and Fe are also found [60]. The aqueous extract of *Channa striata* contains not only amino acid but also palmitic acid (C16 : 0) content [61]. *Channa striata* extract has concentration-dependent antinociceptive activity [61], and *Channa striata* extract takes important roles in recovery from hypoalbuminemia that is commonly found in malnutrition [60]. The nutrient compositions of *Channa striata* extract also elevate the synthesis of collagen fiber during the process of wound healing [59, 60]. Moreover, *Channa striata* supplementation has reduced the availability of *Mycobacterium tuberculosis* significantly among tuberculosis patients during a month [25].

The limitation of this study is the authors cannot describe the effect of *Channa striata* extract on BMI among tuberculosis patients by molecular approach. Therefore, further study is needed to provide information about the relationship between *Channa striata* supplementation and BMI among tuberculosis patients by molecular aspect in order to reduce the duration of standard tuberculosis treatment. In addition, several literature studies noted that BMI is not sensitive enough to detect malnutrition in TB patients.

## 4. Conclusions

The BMI among tuberculosis patients administered *Channa striata* is increasing faster than the control group during a month, and the alteration from severe thinness to higher BMI level in the treatment group is high compared to the control group, although it is not significant by statistical test (*p* > 0.05). In addition, the health status among the treatment group is similar to the control group (*p* > 0.05), except vomiting (*p* < 0.05). Therefore, further study related to the benefit of *Channa striata* extract's utilization toward tuberculosis patients is needed in order to either eliminate or reduce the incidence of tuberculosis in community.

## Figures and Tables

**Table 1:** The respondent characteristics.

Characteristic	All participants (*n* = 200)	Group		*p* value^*∗*^
		Treatment (*n* = 103)	Control (*n* = 97)	
Age, years				0.698
<20	16 (8.00)	9 (8.74)	7 (7.22)	
20–29	37 (18.50)	21 (20.39)	16 (16.49)	
30–39	29 (14.50)	12 (11.65)	17 (17.53)	
40–49	43 (21.50)	24 (23.30)	19 (19.59)	
>50	75 (37.50)	37 (23.30)	38 (39.18)	

Sex				0.197
Male	104 (52.00)	49 (47.57)	55 (56.70)	
Female	96 (48.00)	54 (52.43)	42 (43.30)	

Education				0.313
None	37 (18.50)	18 (17.48)	19 (19.59)	
Elementary	76 (38.00)	37 (35.92)	39 (40.21)	
Junior high school	37 (18.50)	22 (21.36)	15 (15.46)	
Senior high school	47 (23.50)	26 (25.24)	21 (21.65)	
University	3 (1.50)	0 (0.00)	3 (3.09)	

Values are presented as number (%). ^*∗*^Chi-square test. *p* values compared between the treatment and control groups.

**Table 2:** Health status among respondents.

Effect	All participants (*n* = 185)	Group		*p* value^*∗*^
		Treatment (*n* = 94)	Control (*n* = 91)	
Allergy				0.389
None	108 (58.38)	58 (61.70)	50 (54.95)	
Mild	63 (34.05)	38 (29.79)	35 (38.46)	
Moderate	13 (7.03)	8 (8.51)	5 (5.49)	
Severe	1 (0.54)	0 (0.00)	1 (1.10)	

Rash				0.815
None	150 (81.08)	75 (79.79)	75 (82.42)	
Mild	32 (17.30)	17 (18.09)	15 (16.48)	
Moderate	3 (1.62)	2 (2.13)	1 (1.10)	

Inhale nuisance				0.518
None	142 (76.76)	75 (79.79)	67 (73.63)	
Mild	30 (16.22)	13 (13.83)	17 (18.68)	
Moderate	12 (6.49)	5 (5.32)	7 (7.69)	
Severe	1 (0.54)	1 (1.06)	0 (0.00)	

Nausea				0.635
None	110 (59.46)	55 (58.51)	55 (60.44)	
Mild	58 (31.35)	30 (31.91)	28 (30.77)	
Moderate	10 (5.41)	4 (4.26)	6 (6.59)	
Severe	7 (3.78)	5 (5.32)	2 (2.20)	

Vomiting				0.013
None	162 (87.57)	78 (82.98)	84 (92.31)	
Mild	18 (9.73)	14 (14.89)	4 (4.40)	
Moderate	3 (1.62)	0 (0.00)	3 (3.30)	
Severe	2 (1.08)	2 (2.13)	0 (0.00)	

Diarrhea				0.351
None	161 (87.03)	79 (84.04)	82 (90.11)	
Mild	23 (12.43)	14 (14.89)	9 (9.89)	
Moderate	1 (0.54)	1 (1.06)	0 (0.00)	

Smoothing inhale				0.901
None	32 (17.30)	18 (19.15)	14 (15.38)	
Mild	80 (43.24)	40 (42.55)	40 (43.96)	
Moderate	40 (21.62)	19 (20.21)	21 (23.08)	
High	33 (17.84)	17 (18.09)	16 (17.58)	

Good appetite				0.270
None	21 (11.35)	11 (11.70)	10 (10.99)	
Mild	68 (36.76)	37 (39.36)	31 (34.07)	
Moderate	55 (29.73)	22 (23.40)	33 (36.26)	
High	41 (22.16)	24 (25.53)	17 (18.68)	

Values are presented as number (%). ^*∗*^Chi-square test. *p* values compared between the treatment and control groups.

**Table 3:** *Channa striata* supplementation and body mass index at weeks 0–4.

Body mass index	All participants (*n* = 200)	Group		*p* value^*∗*^
		Treatment (*n* = 103)	Control (*n* = 97)	
Week 0				0.625
Severe thinness	70 (35.00)	40 (38.83)	30 (30.93)	
Moderate thinness	28 (14.00)	13 (12.62)	15 (15.46)	
Mild thinness	47 (23.50)	25 (24.27)	22 (22.68)	
Normal	52 (26.00)	23 (22.33)	29 (29.90)	
Overweight	3 (1.50)	2 (1.94)	1 (1.03)	

Week 1				0.705
Severe thinness	62 (31.00)	36 (34.95)	26 (26.80)	
Moderate thinness	32 (16.00)	15 (14.56)	17 (17.53)	
Mild thinness	39 (19.50)	20 (19.42)	19 (19.59)	
Normal	64 (32.00)	30 (29.13)	34 (35.05)	
Overweight	3 (1.50)	2 (1.94)	1 (1.03)	

Week 2				0.501
Severe thinness	56 (28.00)	33 (32.04)	23 (23.71)	
Moderate thinness	31 (15.50)	16 (15.53)	15 (15.46)	
Mild thinness	40 (20.00)	20 (19.42)	20 (20.62)	
Normal	69 (34.50)	31 (30.10)	38 (39.18)	
Overweight	4 (2.00)	3 (2.91)	1 (1.03)	

Week 3				0.726
Severe thinness	54 (27.00)	30 (29.13)	24 (24.74)	
Moderate thinness	30 (15.00)	17 (16.50)	13 (13.40)	
Mild thinness	35 (17.50)	17 (16.50)	18 (18.56)	
Normal	75 (37.50)	35 (33.98)	40 (41.24)	
Overweight	6 (3.00)	4 (3.88)	2 (2.06)	

Week 4				0.923
Severe thinness	47 (23.50)	26 (25.24)	21 (21.65)	
Moderate thinness	31 (15.50)	17 (16.50)	14 (14.43)	
Mild thinness	36 (18.00)	17 (16.50)	19 (19.59)	
Normal	79 (39.50)	39 (37.86)	40 (41.24)	
Overweight	7 (3.50)	4 (3.88)	3 (3.09)	

Values are presented as number (%). ^*∗*^Chi-square test. *p* values compared between the treatment and control groups.

**Table 4:** Body mass index (kg/m^2^) among the treatment and control groups at weeks 0–4.

Group	Time					*p* value^*∗*^
	Week 0	Week 1	Week 2	Week 3	Week 4	
Treatment						0.000
Mean	17.43	17.65	17.90	18.04	18.22	
95% CI	16.82–18.04	17.05–18.24	17.28–18.47	17.44–18.64	17.62–18.82	
SD	3.12	3.06	3.05	3.06	3.07	
IQR	3.84	3.52	3.74	3.55	3.61	
Q1	15.23	15.59	15.82	16.01	16.20	
Q3	19.07	19.11	19.56	19.56	19.81	
Control						0.000
Mean	17.20	17.36	17.57	17.71	17.96	
95% CI	16.70–17.71	16.84–17.88	17.03–18.10	17.03–18.10	17.43–18.49	
SD	2.52	2.58	2.66	2.65	2.62	
IQR	3.29	3.48	3.35	3.40	3.56	
Q1	15.40	15.42	15.70	15.70	15.82	
Q3	18.69	18.90	19.05	19.10	19.38	

^*∗*^Wilcoxon signed-rank test. CI: confidence interval; SD: standard deviation; IQR: interquartile range; Q1: quartile 1; Q3: quartile 3.

## Data Availability

The data used to support the findings of this study are available from the corresponding author upon request.
